# A network-based study reveals multimorbidity patterns in people with type 2 diabetes

**DOI:** 10.1016/j.isci.2023.107979

**Published:** 2023-09-20

**Authors:** Zizheng Zhang, Ping He, Huayan Yao, Renjie Jing, Wen Sun, Ping Lu, Yanbin Xue, Jiying Qi, Bin Cui, Min Cao, Guang Ning

**Affiliations:** 1 Department of Endocrine and Metabolic Diseases, Shanghai Institute of Endocrine and Metabolic Diseases, Ruijin Hospital, Shanghai Jiao Tong University School of Medicine, Shanghai, China; 2 Shanghai National Clinical Research Center for Metabolic Diseases, Key Laboratory for Endocrine and Metabolic Diseases of the National Health Commission of the PR China, Shanghai Key Laboratory for Endocrine Tumor, State Key Laboratory of Medical Genomics, Ruijin Hospital, Shanghai Jiao Tong University School of Medicine, Shanghai, China; 3 Link Healthcare Engineering and Information Department, Shanghai Hospital Development Center, Shanghai, China; 4 Wonders Information Co. Ltd., Shanghai, China; 5 Computer Net Center, Ruijin Hospital, Shanghai Jiao Tong University School of Medicine, Shanghai, China

**Keywords:** Endocrinology, Pathophysiology, Medical informatics

## Abstract

Patients with type 2 diabetes mellitus (T2DM) are at a heightened risk of living with multiple comorbidities. However, the comprehension of the multimorbidity characteristics of T2DM is still scarce. This study aims to illuminate T2DM’s prevalent comorbidities and their interrelationships using network analysis. Using electronic medical records (EMRs) from 496,408 Chinese patients with T2DM, we constructed male and female global multimorbidity networks and age- and sex-specific networks. Employing diverse network metrics, we assessed the structural properties of these networks. Furthermore, we identified hub, root, and burst diseases within these networks while scrutinizing their temporal trends. Our findings uncover interconnected T2DM comorbidities manifesting as emergence in clusters or age-specific outbreaks and core diseases in each sex that necessitate timely detection and intervention. This data-driven methodology offers a comprehensive comprehension of T2DM’s multimorbidity, providing hypotheses for clinical considerations in the prevention and therapeutic strategies.

## Highlights

• A research integrating network analysis, epidemiology, and clinical medicine
• Networks afford a global understanding of the interrelationships among comorbidities
• Mining comorbidity patterns and characteristics from a large-scale clinical dataset
• Identified three categories of crucial diseases can inform clinical decision-making

## Graphical abstract

**Figure:**
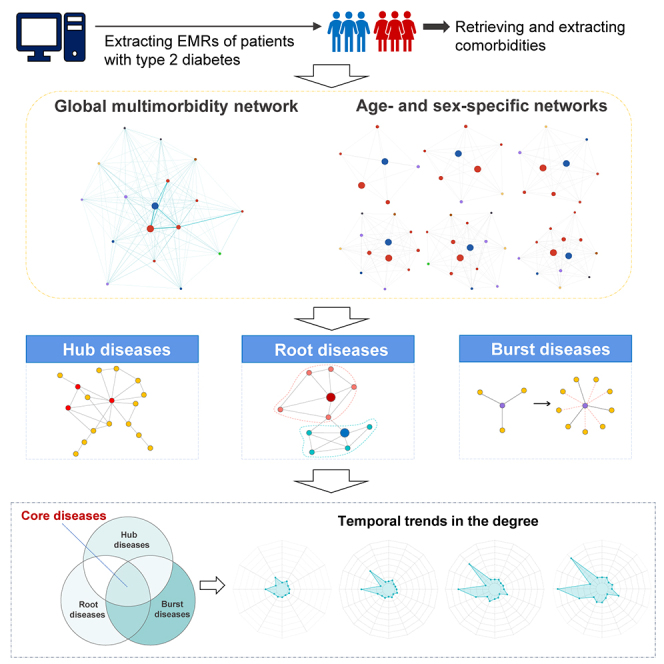

## Introduction

The global prevalence of diabetes mellitus (DM) has dramatically increased, exhibiting a more than 4-fold escalation since 1980, surging from an estimated 108 million to an astounding 463 million as of 2019. Projections indicate that by 2030 this figure is expected to ascend to 578 million, with type 2 diabetes mellitus (T2DM) constituting over 90% of the total cases.^1^^,^^2^ Individuals with DM exhibit a markedly heightened susceptibility to developing comorbidities than those without the condition.^3^ In addition to DM-related complications, individuals with DM commonly experience "concordant" comorbidities, Which represent parts of the same overall pathophysiologic risk profile. These include hypertension, dyslipidemia, and chronic vascular diseases, constituting primary focal points in diabetes care.^4^ Furthermore, "discordant" comorbidities, which lack a pathogenesis connection with DM, have also been frequently identified in patients with DM. Examples of these include depression and thyroid disorders.^5^^,^^6^ Significantly, patients with diabetes rarely encounter solitary comorbidities; instead, they frequently present with concurrent multiple comorbidities, leading to multimorbidity. An investigation conducted in the United States, utilizing an Electronic Medical Record (EMR) database, revealed that 88.5% of individuals with T2DM had a minimum of two comorbidities, with the comorbidity burden intensifying with age.^7^ Likewise, a study conducted in Japan revealed that merely 6.6% of patients with T2DM exhibited a single comorbidity. In contrast, as many as 43.0% presented with four or more comorbidities.^8^ The presence of multimorbidity in patients with diabetes presents substantial challenges to both diabetes care and the healthcare system. These consequences include a diminished quality of life, premature mortality, heightened complexity of medication usage within multimorbidity, and an escalated demand for healthcare resources.^9^^,^^10^^,^^11^

Multimorbidity among patients with diabetes underscores their distinct medical requirements and tailored management strategies. Embracing a patient-centered approach to multimorbidity management necessitates a comprehensive grasp of the patterns in comorbidity. Despite the significance of this issue, research on multimorbidity in individuals with T2DM remains limited. Most studies predominantly centered on investigating the prevalence of comorbidities and comorbidity pairs or utilized cluster analysis to discern comorbidity clusters.^7^^,^^12^^,^^13^ However, multimorbidity should be considered holistically, as diseases can be interconnected.^14^ Network analysis has emerged as a valuable instrument for examining complex comorbid relationships. A multimorbidity network consists of nodes symbolizing diseases and edges signifying the co-occurrence between them. Through analyzing the network, researchers can quantitatively assess direct or indirect relationships between comorbidities, detect clusters comprising conditions that frequently co-occur, and unveil influential diseases that substantially contribute to the burden of multimorbidity. As a rapidly expanding branch of interdisciplinary research, the multimorbidity network has been integrated with machine learning for application in disease risk prediction in recent years, showcasing promising performance.^15^^,^^16^ In studies concerning T2DM, Arif Khan was the pioneering researcher to employ network analysis in the comorbidities of patients with T2DM. His work revealed that the multimorbidity experienced by these patients exhibited greater complexity when compared to non-diabetic individuals.^17^ A Spanish study employed directed networks to assess the comorbidity progression in patients with T2DM and quantified the interconnections among these comorbidities.^18^ While these network-based studies have provided valuable insights into multimorbidity among patients with diabetes, they have predominantly centered on chronic diseases. Given the intricate nature of multimorbidity within patients necessitating prolonged care and its comprehensive influence on their well-being, the European General Practice Research Network has extended the scope of multimorbidity to include the coexistence of chronic and acute conditions.^19^ Furthermore, most studies have been limited by the unavailability of outpatient treatment data, relying on hospital discharge records, which may hamper a global understanding of multimorbidity. Moreover, although sex differences in multimorbidity have been evidenced within the general population,^20^ an investigation into these differences among patients with diabetes remains unexplored. Addressing these research gaps necessitates a comprehensive assessment of multimorbidity in individuals with T2DM, utilizing large-scale clinical data.

In this study, EMRs from 35 hospitals in Shanghai were utilized to construct sex-specific multimorbidity networks for patients with T2DM. Our study’s principal objectives are identifying three categories of crucial diseases within the multimorbidity networks and exploring their temporal trends and sex differences. Specifically, we identified hub diseases that exert a dominant influence on multimorbidity, root diseases that hold the most substantial impact within comorbidity clusters, and burst diseases associated with more comorbidities within specific age ranges. These diseases serve as critical indicators of potential multimorbidity, offering guidance for prioritizing comorbidity management and facilitating the formulation of early prevention strategies within clinical practice. Our study underscores the potential of understanding multimorbidity holistically to improve clinical decision-making and patient care standards for the diabetic population.

## Results

### The characteristics of male and female multimorbidity networks

**Figure 1:**
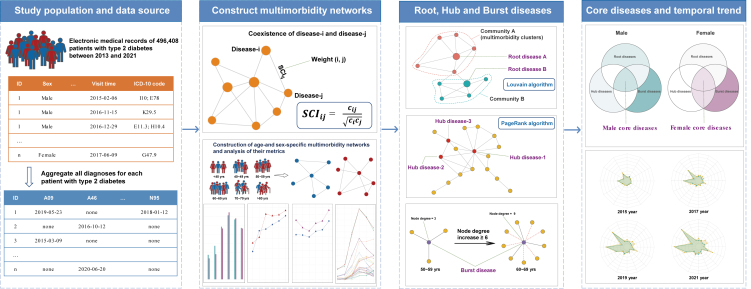
Study workflow SCI: Sclton cosine index.

**Figure 2:**
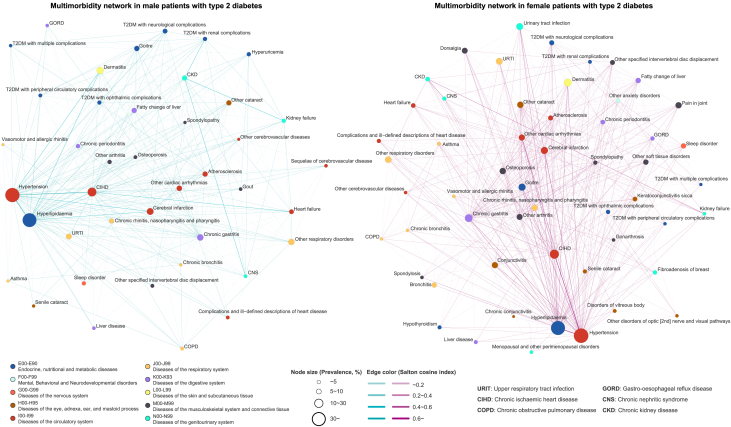
Multimorbidity network in male and female patients with type 2 diabetes Only nodes with a degree greater than or equal to 10 are shown.

The study’s workflow is illustrated in Figure 1. The SCI of any pair of diseases among 177 diseases was calculated, and edges with SCI below the SCI cut-off were excluded. Subsequently, a multimorbidity network was constructed, consisting of 132 nodes and 697 edges, derived from male patients with T2DM. Additionally, a network comprising 144 nodes and 868 edges was created from the female patients. To facilitate visual identification, we displayed sparse multimorbidity networks comprising nodes with a minimum node degree of 10 (Figure 2). We observe that essential hypertension (I10) and the disorders of lipoprotein metabolism and other lipidemias (E78) exhibit the highest frequency of co-occurrence with other diseases. Additionally, circulatory and digestive system diseases also demonstrate more connections. The metrics of the networks are summarized (see Table S2). Notably, the density, average degree, average weighted degree, and average closeness centrality of the multimorbidity network are higher in females than males. Figure S1 shows the heatmap of the network matrix. It reveals a pattern of diseases tending to coexist with others within the same system; diseases of the endocrine & metabolic, and circulatory systems also coexist more with diseases of other systems, particularly in females. Additionally, female patients demonstrate a greater number of coexisting disease pairs within the endocrine & metabolic, musculoskeletal, and genitourinary systems compared to their male counterparts.

### Age- and sex-specific multimorbidity network characteristics

**Figure 3:**
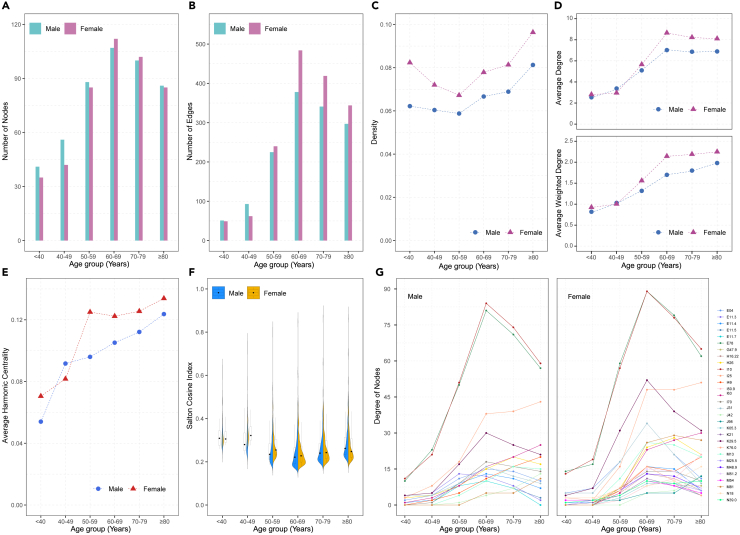
The characteristics of the age- and sex-specific multimorbidity networks (A and B) The numbers of nodes and edges in each multimorbidity network across age strata and by sex. (C–E) The density, average degree, average weighted degree and average harmonic centrality of each multimorbidity network across age strata and by sex. (F) Violin plot of the Sclton cosine index (SCI) of disease pairs across age strata and by sex. (G) The age-based trajectories of the degree of the burst disease in males and females.

Figure 3 illustrates the characteristics of the age- and sex-specific multimorbidity networks. The distribution of the number of nodes and edges of the networks exhibit a “bell-shaped” pattern, with the 60–69 age group network having the highest count of nodes and edges. In the age group under 49, the male networks have more nodes and edges compared to their female counterparts, while the female networks surpass those of males in terms of edge count after the age of 49 (Figures 3A and 3B). Moreover, the trend of the network density displays a “U-shape” pattern in both sexes, with the female networks consistently maintaining a higher density than their male counterparts across all age groups (Figure 3C). The average weighted degree and harmonic centrality of the networks demonstrate an increase with age, while the average degree of the networks constructed from patients over 70 exhibits a slight decrease (Figures 3D and 3E). The SCI of coexisting disease pairs was slightly higher in the lower age group, with a more concentrated distribution than in the higher age group (Figure 3F). Table S3 presents the Top 10 comorbidity pairs with the highest SCI in different age groups among the patients. Essential hypertension (I10) and the disorders of lipoprotein metabolism and other lipidemias (E78) are the most prominently correlated comorbidity pair across all age groups, with the association strength being greater in males than in females up to the age of 59. However, the opposite is true over the age of 59 years. Furthermore, the comorbidity pair consisting of malignant neoplasm of liver and intrahepatic bile ducts (C22) is observed in male patients aged 40 to 69 years.

### Three categories of crucial diseases and temporal trends

Most burst diseases emerge within the 50–69 age group, with female burst diseases demonstrating a more substantial degree increase compared to males. Notably, essential hypertension (I10) and disorders of lipoprotein metabolism and other lipidemias (E78) are the two diseases that exhibit a significant increase in degree (Figure 3G). The age at which burst diseases first appear differs between male and female patients with T2DM. Disorders of lipoprotein metabolism and other lipidemias (E78) emerge as a burst disease in males aged 40–49, occurring earlier than in females at 50–59 (see Tables S4 and S5).

By employing community detection, we have identified six multimorbidity communities in males and seven in females. The root disease for each community was determined based on the maximum eigenvector centrality (Table 1). Among the root diseases of the male network’s community, three out of six belong to the circulatory system. In contrast, the communities in the female network display a greater diversity in terms of their root diseases. The largest communities for both sexes encompass diseases from almost all systems (Community M1 and F1 in Table 1). Nevertheless, they differ regarding their root disease. In males, the root disease is essential hypertension (I10), while in females, it is disorders of lipoprotein metabolism and other lipidemias (E78). Two pairs of communities with the same root disease are observed. However, in the community where chronic ischemic heart disease (CIHD; I25) serves as the root disease, the composition varies: males include only circulatory and respiratory system diseases, whereas females have diseases from eight different systems. Additionally, in the community centered on the other cataract (H26), females have more ocular-related diseases than males. Notably, in the female network, we find the Community F3 consisting of depressive episode (F32) and a range of psychiatric-related disorders, with other anxiety disorders (F41) serving as the root disease. In contrast, in males, these illnesses were assigned to the Community M1, centered on essential hypertension (I10). Moreover, males do not exhibit multimorbidity communities with other arthritis (M13) and other nontoxic goiter (E04) as the root disease compared to females.

**Table 1:** The community in multimorbidity networks in male and female patients with type 2 diabetes

Communities (Nodes-Edges)	Root disease	Member withnin the community
M1 (65–216)	hypertension (I10)	A49, B02, B35, B36, C61, E78, F32, F41, F51.9, G47.9, H10.4, H10.9, H11, H16.22, H60.9, H93.1, I10, I83, J02, J06, J15, J18.9, J20, J30, J31, J32, J37, K02, K04, K05.3, K21, K25.9, K29.3, K29.4, K29.5, K29.6, K29.7, K30, K52.9, K59.9, K63, K81.9, K92, L08, L21, L23, L29, L30.9, L40, L50, M13, M17, M19, M25.5, M47, M48.0, M48.9, M50.2, M51.2, M54, M79, M81, N13, N39.0, N39.9
M2 (8–19)	CKD (N18)	D64.9, E11.2, E79.0, E87, M10, N03, N18, N19
M3 (18–55)	CIHD (I25)	I20, I21, I24, I25, I44, I48, I49, I50.9, I51, J40, J42, J43, J44.9, J45.9, J47, J94, J96.9, J98
M4 (9–16)	Fibrosis and cirrhosis of liver (K74)	B18.1, C18, C22, C34, C78, K72.9, K73, K74, K76.9
M5 (25–58)	Atherosclerosis (I70)	C73, E03.9, E04, E05, E06, E11.4, E11.5, E11.7, E66.9, F03, G20, G31, G45, G62, I63, I65, I67, I69, I70, K76.0, K76.8, K80.2, L28, N20, N28.1
M6 (7–19)	Other cataract (H26)	E11.3, H25, H26, H35, H40.9, H43, H47
F1 (51–124)	Hyperlipidemia (E78)	A09, A49, B02, B07, B35, C34, C50, E78, G20, H11, H81.9, H93.1, I10, I83, J15, J18.9, J20, K02, K04, K12, K21, K25.9, K29.3, K29.4, K29.5, K29.6, K29.7, K30, K52.9, K59.9, K63, K80.2, K81.9, K92, L08, L21, L23, L29, L50, M06, M65, M75, N20, N39.9, N63, N64, N76.0, N60.2, N76.1, N92, N95
F2 (34–125)	CIHD (I25)	D64.9, E11.2, E79.0, E87, F03, G31, G45, I20, I21, I24, I25, I44, I48, I49, I50.9, I51, I63, I65, I67, I69, I70, J40, J94, J96.9, J98, J42, J44.9, J45.9, J47, M10, N03, N18, N19, N39.0
F3 (5–6)	Other anxiety disorders (F41)	F32, F41, F48, F51.9, G47.9
F4 (10–28)	Other cataract (H26)	H10.9, E11.3, H10.4, H16.22, H25, H26, H35, H40.9, H43, H47
F5 (22–72)	Other arthritis (M13)	H60.9, J02, J06, J30, J31, J32, J37, K05.3, L30.9, M13, M15, M17, M19, M25.5, M47, M48.0, M48.9, M50.2, M51.2, M54, M79, M81
F6 (8–11)	Chronic hepatitis, not elsewhere classified (K73)	B18.1, C18, C22, C78, K72.9, K73, K74, K76.9
F7 (14–25)	Other nontoxic goiter (E04)	C73, E03.9, E04, E05, E06, E11.4, E11.5, E11.7, E66.9, G62, L28, K76.0, K76.8, N28.1

M1-M6: Male Communities; F1-F7: Female communities.

CKD: Chronic kidney disease.

CIHD: Chronic ischemic heart disease.

**Figure 4:**
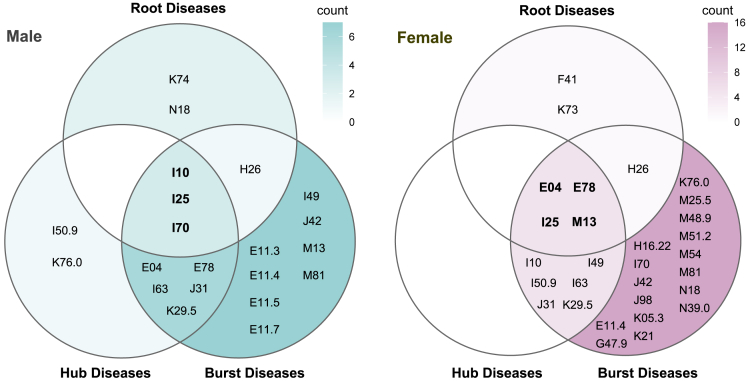
The root, hub, and burst diseases in male and female patients with type 2 diabetes The intersection of the three circles, the central area, is the core diseases.

Figure 4 depicts the three categories of crucial diseases determined from the multimorbidity network constructed by male and female patients with T2DM. Among the ten hub diseases for each sex, eight are shared by both males and females. The male-specific hub diseases are atherosclerosis (I70) and nonalcoholic fatty liver disease (NAFLD; K76.0), while the female-specific ones are other cardiac arrhythmias (I49) and other arthritis (M13). Furthermore, half of the ten hub diseases in both sexes belong to the circulatory system. Regarding the burst disease, there are 27 types in females and 17 in males. Remarkably, the burst diseases in females contain more musculoskeletal and digestive system diseases. Conversely, males have four DM-related complications (E11.3, E11.4, E11.5, and E11.7), whereas females have only one. Ultimately, three core diseases for males and four for females were determined. The core diseases in males are all from the circulatory system: essential hypertension (I10), CIHD (I25), and atherosclerosis (I70). The core diseases for females from three different disease systems are other nontoxic goiter (E04), disorders of lipoprotein metabolism and other lipidemias (E78), CIHD (I25), and other arthritis (M13).

**Figure 5:**
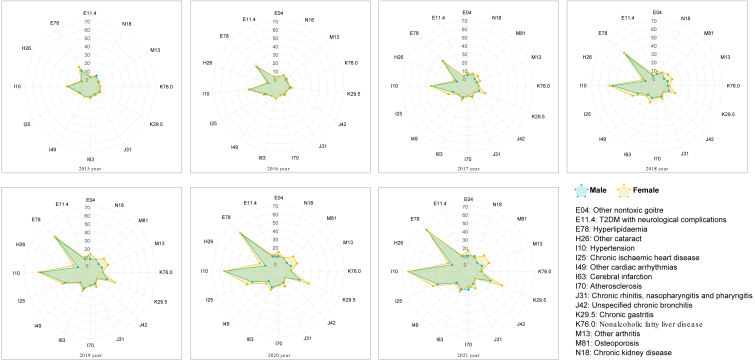
The temporal trends in the degree increase of the root, hub, and burst diseases shared by both sexes There were 12 and 15 in 2015 and 2016, respectively, and 16 in all the remaining years.

We identified 16 diseases that fell within the three categories of crucial diseases shared by both sexes and observed their trends in the degree increase (Figure 5). Notably, all of these diseases exhibit a consistent increase in the degree for seven years starting from 2015. Among them, essential hypertension (I10) and disorders of lipoprotein metabolism and other lipidemias (E78) display particularly noteworthy increases in the degree. Additionally, females exhibit more significant degree increases in other nontoxic goiter (E04), other arthritis (M13), osteoporosis without current pathological fracture (M81), CIHD (I25), and unspecified chronic gastritis (K29.5) than males, and these diseases overlap highly with their core diseases.

### Perturbation analyses

The results of the perturbation analyses substantiated the influence exerted by the 16 identified crucial diseases on multimorbidity patterns and assessed the robustness of the global networks (see Tables S6 and S7). Specifically, the removal of any of these crucial diseases impacts the network’s topology. Among male patients, the elimination of disorders of lipoprotein metabolism and other lipidemias (E78), as well as circulatory system diseases, significantly diminishes the network size and complexity of multimorbidity. Remarkably, the exclusion of essential hypertension (I10) induces a remarkable 6.744% reduction in the network’s average harmonic centrality, surpassing the observed 2.722% reduction in females. Conversely, within the female patients, the absence of other nontoxic goiter (E04), unspecified chronic gastritis (K29.5), and musculoskeletal system diseases exhibit a more pronounced influence on the structural characteristics of the networks compared to their male counterparts. Furthermore, the omission of disorders of lipoprotein metabolism and other lipidemias (E78) in the female network results in a reduction of 4.593% in the average harmonic centrality, markedly exceeding the 1.885% reduction observed in males. These observations closely align with the respective core diseases identified in male and female patients, bolstering our findings' credibility.

The random deletion of network edges exhibits a relatively minimal impact on the network’s critical nodes (see Table S8). Upon implementing three levels of edge removal for both male and female multimorbidity networks, the hub diseases demonstrate only minor alterations in their ranking sequence, with a single hub disease introduced to each sex: other cardiac arrhythmias (I49) for males and osteoporosis without current pathological fracture (M81) for females. Notably, even with the elimination of 5% of edges, the integrity of 90% of the hub diseases remains unaltered. These findings provide substantiation for the robustness of the multimorbidity networks constructed.

## Discussion

We employed a network-based approach to analyze comorbidities in the Chinese population with T2DM, aiming to comprehend their complex interconnections. The multimorbidity network offered a global picture of comorbidities, revealing insights that transcend isolated investigations of singular comorbidities. Our results provided a detailed characterization of multimorbidity in Chinese adults with T2DM and determined a specific cluster of comorbidities that necessitate heightened attention within the medical care of patients with diabetes.

In the visualization of the multimorbidity networks, essential hypertension (I10) and disorders of lipoprotein metabolism and other lipidemias (E78) were the most conspicuous nodes, demonstrating the most connections with other comorbidities, which seems to be the reflection of the metabolic syndrome (Mets) in the disease network. The Mets is a group of cardiovascular disease (CVD) risk factors centered on disorders of glucose metabolism, hypertension, dyslipidemia, and abdominal obesity.^21^ A multicenter study conducted in China reported that up to 68.1% of newly diagnosed individuals with T2DM had Mets.^22^ This percentage may be even higher considering that our study population was not limited to newly diagnosed patients with T2DM. Mets-related oxidative stress and chronic inflammation elevate the risk of CVD and all-cause mortality. Furthermore, they may predispose patients to multiple comorbidities, exhibiting their coexistence with hypertension and dyslipidemia.^23^^,^^24^^,^^25^ CVD continues to be the primary cause of mortality among patients with T2DM. Hypertension and dyslipidemia are recognized as pivotal modifiable risk factors for CVD.^26^^,^^27^^,^^28^ Prior studies have underscored the significant advantages of improving lipid and blood pressure levels in patients with T2DM to reduce the risk of CVD.^29^^,^^30^^,^^31^^,^^32^ The combined management of blood pressure, lipid levels, and glucose has been shown to significantly decrease the risk of comorbidities in patients with T2DM and enhance their quality of life.^33^^,^^34^ Our findings further underscore the significance of combined management for them when considering the burden of multimorbidity.

Global multimorbidity network metrics indicated that in female patients with T2DM, both the complexity of multimorbidity and the strength of comorbidity associations were higher than in males. While previous studies have not explored sex differences in comorbidity interconnections among patients with diabetes, our findings align with observations observed in the general population.^20^ Among patients with T2DM, the most expansive spectrum of multimorbidity was observed in the age range of 60–69, while this extent exhibited a minor decline in older age groups. This phenomenon might be attributed to survivorship bias, as patients with diabetes with more comorbidities face an elevated mortality risk. A previous study has substantiated the significant correlation between an escalation in the number of comorbidities in patients with T2DM and all-cause mortality.^35^ We discovered that expansion in the multimorbidity spectrum did not consistently correlate with the increase in comorbidity pairs, as evidenced by the “U-shaped” networks’ density. Nevertheless, the rapid growth of the average weighted degree and edge count within the networks from 40 to 69 years of age could elucidate this phenomenon. Younger patients with T2DM suffer from more prevalent but less numerous comorbidities closely interconnected with each other. Many comorbidities emerge with advancing age or prolonged duration of T2DM, yet these are not universally interrelated. As the cumulative strength and count of associations among various comorbidities increased, the patients became more susceptible. This susceptibility could foster the formation of new coexisting comorbidity pairs or the transformation of previously indirectly linked comorbidity pairs into directly linked ones. As a result, the networks’ density gradually rises again. The proportion of patients with diabetes with three or more comorbidities increases from 30% to 60% ten years after diagnosis,^36^ which may support the notion.

The patients with T2DM in the lower age group exhibited the lowest number but higher strength of comorbidity pairs. Among the female patients under 49 years of age, essential hypertension (I10) and disorders of lipoprotein metabolism and other lipidemias (E78) were more likely to coexist with unspecified chronic gastritis (K29.5), unspecified dermatitis (L30.9), and excessive, frequent, and irregular menstruation (N92). In contrast, male patients under 49 had more comorbidity pairs composed of DM-related ophthalmic complications (E11.3) and ocular diseases. The UK Prospective Diabetes Study discovered that the male sex constitutes an independent risk factor for more severe diabetic retinopathy and its progression in patients with diabetes.^37^ Significantly, the association strength of fibrosis and cirrhosis of the liver (K74) and malignant neoplasm of the liver and intrahepatic bile ducts (C22) ranked high among male patients aged 40 to 69. Additionally, among male patients aged 40 to 49, the strength of association between nonalcoholic fatty liver disease (NAFLD; K76.0) and essential hypertension (I10) or disorders of lipoprotein metabolism and other lipidemias (E78) was also remarkably significant. T2DM and NAFLD frequently co-occur and synergistically contribute to increased risk of adverse outcomes. Moreover, T2DM also serves as a risk factor for the development of cirrhosis and hepatocellular carcinoma in individuals with NAFLD.^38^^,^^39^ Our findings emphasize the significance of promptly identifying and addressing NAFLD in young males with T2DM.

The hub diseases hold the most potent influence within the multimorbidity network, coexisting with numerous comorbidities that co-occur with many other diseases. These co-occurring comorbidity pairs may be due to shared genetic or pathological mechanisms, common risk factors, or the progression of one disease contributing to the onset of another.^14^^,^^40^^,^^41^ Based on this principle, the identification and intervention of treatable hub diseases may alleviate the burden of multimorbidity.^42^ Among the ten hub diseases in each network, male and female patients with T2DM shared eight: essential hypertension (I10), CIHD (I25), unspecified heart failure (I50.9), cerebral infarction (I63), disorders of lipoprotein metabolism and other lipidemias (E78), other nontoxic goiter (E04), chronic rhinitis, nasopharyngitis and pharyngitis (J31), and unspecified chronic gastritis (K29.5). Half of these belong to the circulatory system, underscoring the significant impact of circulatory comorbidities in increasing the multimorbidity spectrum in patients with T2DM. We defined the comorbidity as burst diseases when it exhibit an increase in node degree of six or more between adjacent age groups, meaning that it might co-occur with at least six other diseases upon their initial manifestation. Burst diseases were predominantly concentrated within the age range of 50–69, and they were far more numerous in female patients than males. Consequently, the burst diseases hold the potential to serve as a metric for potential multimorbidity burden. Monitoring burst diseases could facilitate the early detection of other diseases, particularly in middle-aged female patients with T2DM.

Communities within multimorbidity networks provide valuable insights into the clustering of comorbidities. Liya Wang found that the number of chronic diseases in the community increased over time, but the new entrants did not replace the root of the community.^43^ In our study, sex influenced the clustering pattern of multimorbidity. Among individuals with T2DM, females exhibited more complex multimorbidity communities and more strongly interconnected psychiatric disorders than males. A distinctive Community F3 emerged among female patients, comprising depressive episodes (F32) as one of its constituents, with other anxiety disorders (F41) serving as its root disease. This observation aligns with prior research, where anxiety plays a vital role in developing and maintaining depression.^44^^,^^45^ Furthermore, depression in patients with diabetes is linked to poor medication adherence and an increased risk of microvascular and macrovascular complications.^46^^,^^47^ Hence, screening and intervention for anxiety symptoms among females with T2DM are critical in promoting their overall psychiatric health. Both biological and psychosocial factors influence the differences in comorbidities of patients with T2DM between sexes.^48^^,^^49^ Further investigation into the sex-dimorphic pathophysiological mechanisms of T2DM and its complications could contribute to advancing personalized diabetes care and awareness regarding sex- and gender-specific risk factors.

The final core diseases identified amalgamated characteristics of hub disease, root disease, and burst disease. Their presence in the patients signifies the potential for multimorbidity outbreaks and the emergence of comorbidity clusters. The sex differences in the core diseases suggest that assessing thyroid and osteoarticular health is imperative for managing multimorbidity in females with T2DM, complementing the traditional focus on CVD risk. Specifically, the prevalence of depression and thyroid-related disorders is higher in females than in males, and female patients with T2DM diagnosed with nontoxic goiter may experience heightened levels of depression due to anxiety.^50^ The clustered appearance of osteoarthritis implies frequent utilization of NSAIDs, which in turn elevate cardiovascular risk.^51^^,^^52^ We also analyzed temporal trends in the three categories of crucial diseases over an extended study period. The count of connections of all these diseases showed a yearly increment, with hypertension and dyslipidemia exhibiting the most substantial increases, suggesting the pivotal role of these two diseases in the progression of multimorbidity among patients with T2DM. Anders Boeck Jensen explored the trajectories of progression for various chronic diseases, including diabetes clusters, and identified several central diseases in the progression of these conditions.^53^ Alba Aguado noticed the relevance of retinopathy in the progression to complicated hypertension, cerebrovascular disease, ischemic heart disease, and organ failure through temporal associations.^18^ These findings underscore the significance of promptly detecting and intervening in the crucial diseases within multimorbidity.

The primary limitations in the multimorbidity studies lie in the methodology employed for collecting disease records and ensuring their completeness. This includes whether medical records are gathered through self-report or physician diagnosis and whether disease records exclusively stem from hospital discharge records or encompass outpatient records. Our study analyzed comorbidity co-occurrence using a comprehensive EMR database that includes inpatient and outpatient information on patients with diabetes, Which allowed us to investigate common comorbidity patterns in T2DM. Furthermore, incorporating comorbidities into the network analysis enables the exploration of both indirect and direct relationships between comorbidities that could not be obtained when studying comorbidities in isolation. As such, network analysis offers a comprehensive picture of multimorbidity in patients with T2DM, yielding new insights into developing management strategies for addressing multimorbidity in this population.

In conclusion, the comorbidities of patients with T2DM were interconnected and manifested as emergence in clusters and age-specific outbreaks. Through our investigation, we have identified a set of crucial diseases that improve comprehension of the complex interconnections within T2DM comorbidities. This knowledge could aid clinicians in anticipating the likelihood of associated diseases of patients with T2DM. Closely monitoring of these diseases and timely intervention could impede their spread or decelerate the progression of more comorbidities, reducing the further burden of multimorbidity. Female and older patients with T2DM were at higher risk of developing multimorbidity. Future research should expand the current findings, considering differences in race, ethnicity, or other covariates to characterize the heterogeneity of T2DM better. Additionally, incorporating molecular and genetic data can contribute to elucidating potential mechanisms underlying the identified associations.

### Limitations of the study

The present study does have certain limitations. While our analysis revealed associations between comorbidities of patients with T2DM, the causality could not be derived, and the reasons for the co-occurrence remained undetermined. Nevertheless, the observed pairwise combinations of morbidities occurred with a frequency more significant than that expected by random chance, which still offers valuable insights for clinical practice. Furthermore, our analysis did not include individual-level socioeconomic status and lifestyle variables. These factors play a significant role in understanding patterns of multimorbidity. Additionally, the study population predominantly consists of Asian individuals. Therefore, caution should be exercised when extrapolating the findings to individuals of other racial backgrounds.

## STAR★Methods

### Key resources table

**Table:** 

REAGENT or RESOURCE	SOURCE	IDENTIFIER
**Deposited data**		

Anonymized electronic medical records	Shanghai Link Healthcare Database	N/A

**Software and algorithms**		

R	R Core Team	https://www.R-project.org/
RStudio	RStudio Team	http://www.rstudio.com/
data.table (R-package)	Open source	https://cran.r-project.org/web/packages/data.table/index.html
tidyverse (R-package)	Open source	https://cran.r-project.org/web/packages/tidyverse/index.html
lubridate (R-package)	Open source	https://cran.r-project.org/web/packages/lubridate/index.html
igraph (R-package)	Open source	https://cran.r-project.org/web/packages/igraph/index.html
ggplot2 (R-package)	Open source	https://cran.r-project.org/web/packages/ggplot2/index.html
cowplot (R-package)	Open source	https://cran.r-project.org/web/packages/cowplot/index.html
ggunchained (R-package)	Open source	https://github.com/jankounchained/ggunchained
gridExtra (R-package)	Open source	https://cran.r-project.org/web/packages/gridExtra/index.html
ggVennDiagram (R-package)	Open source	https://cran.r-project.org/web/packages/ggVennDiagram/index.html
fmsb (R-package)	Open source	https://cran.r-project.org/web/packages/fmsb/index.html
corrplot (R-package)	Open source	https://cran.r-project.org/web/packages/corrplot/index.html
Codes in this paper	This paper	https://github.com/ZhangZizheng-epi/multimorbidity-patterns

### Resource availability

#### Lead contact

Further information and requests for data access should be directed to and will be fulfilled by the lead contact, Bin Cui (cb11302@rjh.com.cn).

#### Materials availability

No materials were used in this study.

### Experimental model and study participant details

The dataset for this study came from the EMRs of 496,408 Chinese T2DM patients aged 18 years and older at the Shanghai Link Healthcare Database (SLHD). There are no restrictions on ethnicity and sex in the current study.

Any personally identifiable information was scrambled to protect privacy. Consequently, the study was exempt from requiring institutional review board approval, as the researchers were blinded to patient identities.

### Method details

#### Data source and study population

The study’s workflow is illustrated in Figure 1. The data employed in this research was sourced from the SLHD. This comprehensive database aggregates general medical practice information for patients across 35 hospitals in Shanghai and covers more than 99% of the city’s residents. Utilizing unique Hospital Link identification cards facilitates access to medical data for each resident, including age, visit time, and diagnoses coded according to the ICD-10 (International Classification of Diseases, 10th revision). The SLHD has been releasing data for academic research since 2013, which requires review and approval to access.

We extracted information on patients with T2DM from the database between January 1, 2013, and December 31, 2021. In order to acquire complete medical diagnosis records for each patient, we included only those individuals who had undergone a minimum of three outpatient visits or at least one hospitalization annually between their initial and final appearance within the database. We defined the index date as the date of the earliest recorded code for T2DM. The patient’s age was calculated as the average age between the index date and the last visit date. Patients with conflicting sex information or missing age or sex information were excluded from our analysis. Finally, the dataset subject to analysis consisted of 255,978 male T2DM patients and 240,430 female T2DM patients, all 18 years of age and older.

#### Measuring multimorbidity

In the context of our research, we define multimorbidity as the concurrent existence of one chronic disease alongside at least one other chronic or acute condition, excluding T2DM, after being diagnosed with T2DM. To examine the influence of the prevailing conditions, we limited our analysis to those with a prevalence of 1% or higher. We only incorporated the ICD-10 codes from chapters 1 to 14, which describe the conditions rather than symptoms or signs. We relied on The Chronic Condition Indicator to differentiate between acute and chronic diseases.^54^ If a three-character ICD-10 code refers to a disease that does not contain both subdivided acute and chronic conditions, it was included in our analysis; otherwise, we included acute and chronic conditions coded by four or more characters, respectively. Ultimately, we identified 177 conditions for analysis (see Table S1).

#### Constructing multimorbidity network

A network is comprised of nodes and edges between them. In our multimorbidity network, nodes correspond to diseases, and edges connect two coexisting diseases. The strength of the edge holds clinical significance, as higher strength signifies an elevated probability of disease co-occurrence. Previous studies employed the phi correlation coefficient or relative risk to quantify this strength.^14^^,^^42^^,^^55^ However, these measures may not be suitable for constructing sparse multimorbidity networks due to their violation of the null invariance property^56^; Specifically, these estimates are susceptible to the total number of observations utilized, which may lead to overestimation or underestimation of the strength and make comparing the networks constructed from different sample sizes challenging. This limitation can impede the understanding of diseases co-occurrence.

The Salton Cosine Index (SCI) is advantageous in constructing multimorbidity networks, as the properties and topology of the resulting network are not influenced by sample size.^57^ This study employed the SCI to construct age- and sex-specific multimorbidity networks in male and female patients. Age was stratified into <40, 40–49, 50–59, 60–69, 70–79, and ≥80 years old.

(Equation 1) $${SCI}_{ij}=\frac{c_{ij}}{\sqrt{c_{i}c_{j}}}$$

(Equation 2) $$\Phi_{ij}=\frac{c_{ij}N-c_{i}c_{j}}{\sqrt{c_{i}c_{j}\left(N-c_{i}\right)\left(N-c_{j}\right)}}$$

(Equation 3) $$t_{ij}=\frac{\Phi_{ij}\sqrt{c_{ij}-2}}{\sqrt{1-{\Phi_{ij}}^{2}}}$$

Where $c_{ij}$ denotes the count of the patients with both disease *i* and disease *j*, $c_{i}$ and $c_{j}$ represent the count of the patients with diseases *i* and *j*, respectively, and *N* is the number of patients. Since negative correlations also lead to positive SCI values, we had to determine a cut-off for the SCI to ensure that our estimates indicated the diseases' co-occurrence. To this end, we employed the relationship between the SCI and the phi coefficient to determine the cut-off. The basic principle of this approach is that the number of positively correlated diseases is equal in the networks constructed using both measures.^58^ The steps to find the cut-off are as follows:

Step 1. For any pair of diseases from 177 diseases, calculate the SCI (calculated in Equation 1) and phi correlation coefficient (Φ, calculated in Equation 2).

Step 2. Find the number of pairs of diseases (*q*), i.e., the count of the pairs with $c_{ij}$ >0.

Step 3.Determine the number of disease pairs (*e*) that satisfy both *p* < 0.01 ($t_{ij}$ >2.58; calculated in Equation 3) and $c_{ij}$ >$\sum c_{ij}/q$.

Step 4. Calculate the SCI cut-off using *e* and construct multimorbidity networks.

### Quantification and statistical analysis

#### Network metrics

We employed a range of network metrics to measure the structural properties of the multimorbidity network, including degree, weighted degree, density, and closeness centrality. The degree of a specific node signifies the count of its direct connections to other nodes, while the weighted degree is the degree calculated considering the weights (SCI) of the edges. Density, the ratio of the actual number of edges within a network to the potential number of edges between all nodes, serves as a metric reflecting the complexity of multimorbidity. Closeness centrality quantifies the proximity of a node to all other nodes within the network. A node possessing higher closeness centrality indicates a stronger interconnectedness with other nodes. Due to the age- and sex-specific multimorbidity networks are not all connected, we have substituted closeness centrality with harmonic centrality, a conceptually akin measure, for their quantification.

#### Hub, root, and burst diseases

Critical nodes refer to those specific nodes within a network that substantially influence its structure. In the context of our study, we identified three categories of crucial diseases from three dimensions, namely hub diseases with potent influence within the entire multimorbidity network, root diseases that are most representative within multimorbidity clusters, and burst diseases that co-occur with a greater number of other diseases within specific age groups.

PageRank algorithm was employed to identify the nodes with strong influence in the network.^59^ The nodes with higher PageRank values significantly influence the entire network. We defined the top 10 nodes with the highest PageRank values as hub diseases within the network. In clinical practice, it is imperative to accord these diseases ample attention, as they may serve as a priority for intervention to reduce the multimorbidity burden or as a signal for pre-multimorbidity.

The community detection algorithm allows for segmenting the multimorbidity network into distinct communities. The nodes within the same community exhibit stronger interconnections than those across different communities. We employed the Louvain algorithm to identify communities within the network. Additionally, we designated the node with the maximum eigenvector centrality within each community as its root disease.^60^ As the most influential node within a community, the root disease can help physicians screen for and prevent other diseases within the same community.

We constructed a total of 12 age- and sex-specific networks. The nodes with a degree growth of ≥6 between adjacent age groups were defined as burst diseases, and we depicted the trajectories of these nodes' degrees. Identifying the age at which burst diseases occur contributes to our comprehension of multimorbidity progression, facilitates the formulation of corresponding preventive strategies, and reinforces multimorbidity awareness and educational efforts among patients in specific age groups.

#### Temporal trends and core diseases

In order to determine the most influential diseases in the context of multimorbidity, we defined the intersection of the three categories mentioned above as core diseases, which should be the most vigilant in managing multimorbidity in T2DM patients. Moreover, we examined the temporal trends in the degree of the three categories of crucial diseases shared by both males and females. To obtain continuous diagnosis records, we specifically selected 51,043 patients who had their initial T2DM code recorded in 2013 and sought medical attention annually from 2013 to 2021. Setting the baseline year as 2015, we construct a multimorbidity network for each subsequent year. For the 2015 network, we extracted diagnoses from 2013 to 2015; for the subsequent years, we encompassed all available medical histories. Ultimately, we employed seven dynamic multimorbidity networks to investigate the temporal trends.

#### Perturbation analyses

Finally, we conducted perturbation analyses on the global multimorbidity networks of male and female T2DM patients. The objective was to validate the impact of the three identified categories of crucial diseases on the networks' structural properties and to assess the networks' robustness. Perturbations were applied to the network nodes by removing one crucial disease at a time, followed by the evaluation of network metrics. Moreover, perturbations were introduced to the network edges through random edge deletions. Concretely, we executed the edge removal at three levels: 1%, 3%, and 5% of total edges, with each level executed thrice. Subsequently, we identified the top 10 diseases with the highest PageRank values in each execution, observing the differences from those in the original network. All statistical analyses and visualization were performed using R, version 4.0.3.

## Data Availability

• The anonymized electronic medical data reported in this study cannot be deposited in a public repository because were used under license for the current study, and so are not publicly available. To request access, contact the corresponding author Bin Cui from Shanghai Institute of Endocrine and Metabolic Diseases. • All original code has been deposited on GitHub: https://github.com/ZhangZizheng-epi/multimorbidity-patterns and is publicly available as of the date of publication. • Any additional information required to reanalyse the data reported in this paper is available from the lead contact upon request.

## Supplemental information

Document S1. Figure S1 and Tables S2–S8

Table S1. diseases and the corresponding ICD-10 codes, related to the STAR Methods
