# Supplementary material for: A network-based study reveals multimorbidity patterns in people with type 2 diabetes
Source: iScience. 2023 Sep 20;26(10):107979. doi: 10.1016/j.isci.2023.107979 (PMC10562779; doi:10.1016/j.isci.2023.107979)
Supplement: Document S1. Figure S1 and Tables S2–S8 [file mmc1.pdf]

iScience, Volume *26*

Supplemental information

A network-based study reveals multimorbidity patterns in people with type 2 diabetes

Zizheng Zhang, Ping He, Huayan Yao, Renjie Jing, Wen Sun, Ping Lu, Yanbin Xue, Jiying Qi, Bin Cui, Min Cao, and Guang Ning

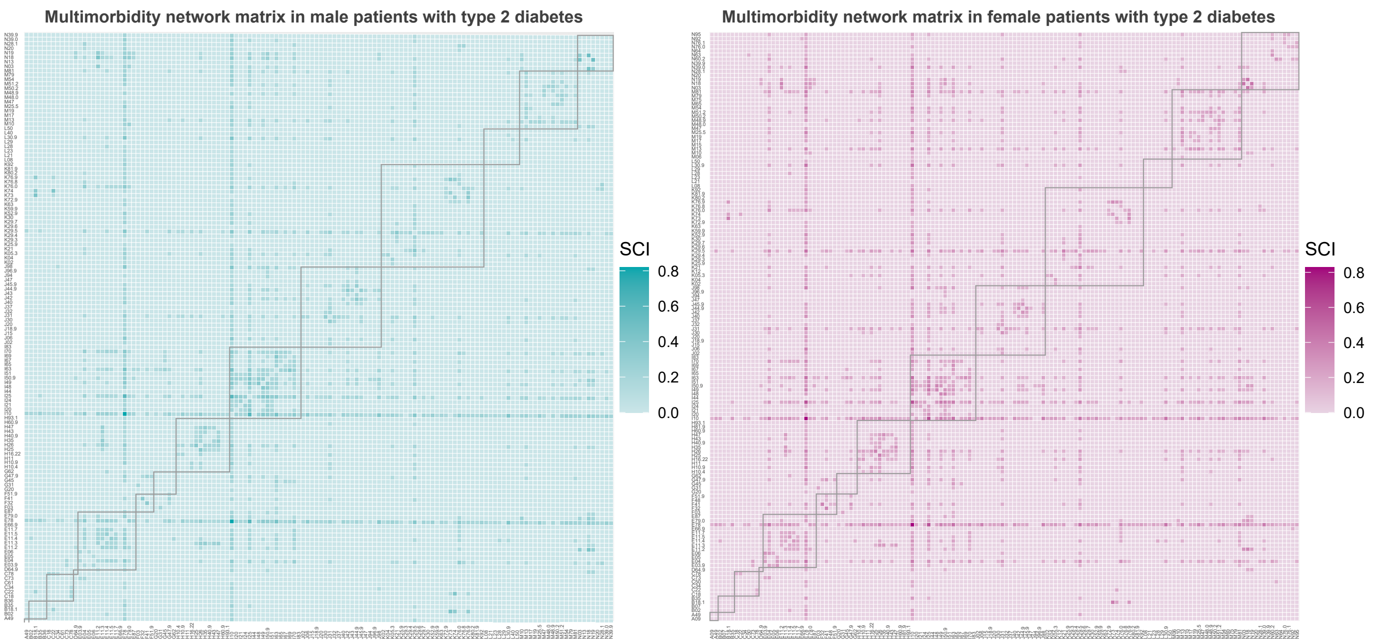

Fig. S1: Multimorbidity network matrix in male patients with type 2 diabetes, related to the STAR Methods.

Within the gray rectangle are relationships between diseases within the same system. SCI: Salton Cosine Index.

TableS2. Metrics of multimorbidity networks in male and female patients with type 2 diabetes, related to the STAR Methods.

**Metrics**	**Male**	**Female**
No.of Patients	255,978	240,430
Nodes (Diseases)	132	144
Edges (Comorbidity pairs)	697	868
Density	0.081	0.084
Average Degree	10.561	12.056
Average Weighted Degree	2.068	2.431
Average Closeness Centrality	0.094	0.099

TableS3. Top 10 comorbidity pairs with the highest Salton Cosine Index (SCI) in different age groups in patients with type 2 diabetes, related to Figure 3.

Sex	<40 yrs	SCI	40~49 yrs	SCI	50~59 yrs	SCI	60~69 yrs	SCI	70~79 yrs	SCI	≥80 yrs	SCI
Male	E78--I10	0.61	E78--I10	0.73	E78--I10	0.80	E78--I10	0.84	E78--I10	0.86	E78--I10	0.86
	E78--K76.0	0.48	B18.1--K73	0.47	E78--I25	0.54	E78--I25	0.62	E78--I25	0.66	E78--I25	0.70
	E11.3--H35	0.40	C22--K74	0.44	C22--K74	0.51	C22--K74	0.47	E78--I63	0.53	E78--I63	0.57
	E11.3--H43	0.40	E11.2--N18	0.43	E11.2--N18	0.48	E11.2--N18	0.46	E11.2--N18	0.45	J98--I10	0.52
	B18.1--K73	0.40	E78--K76.0	0.41	B18.1--K73	0.45	E78--I63	0.46	L30.9--I10	0.44	J98--E78	0.50
	E11.3--H47	0.36	E11.3--H43	0.41	B18.1--K74	0.41	B18.1--K73	0.43	L30.9--E78	0.43	L30.9--I10	0.48
	I10--K76.0	0.36	E78--I25	0.41	L30.9--I10	0.38	L30.9--E78	0.42	J98--I10	0.43	E78--I49	0.47
	J06--I10	0.35	E11.3--H35	0.37	L30.9--E78	0.38	L30.9--I10	0.42	B18.1--K73	0.42	L30.9--E78	0.46
	E11.2--N18	0.33	I10--K76.0	0.36	E78--K29.5	0.37	E78--K29.5	0.40	E78--I49	0.41	E78--I50.9	0.45
	C73--E04	0.33	B18.1--K74	0.36	E78--I63	0.37	I10--K29.5	0.39	J98--E78	0.41	I10--N18	0.41
Female	E78--I10	0.58	E78--I10	0.68	E78--I10	0.79	E78--I10	0.85	E78--I10	0.87	E78--I10	0.86
	E78--N92	0.48	J06--I10	0.44	E78--I25	0.49	E78--I25	0.59	E78--I25	0.67	E78--I25	0.71
	I10--N92	0.42	L30.9--I10	0.41	E78--K29.5	0.46	E78--K29.5	0.50	E78--I63	0.53	E78--I63	0.58
	J06--I10	0.42	J06--E78	0.41	L30.9--I10	0.45	I10--K29.5	0.49	E78--K29.5	0.47	J98--I10	0.50
	J06--E78	0.41	B18.1--K73	0.40	L30.9--E78	0.45	L30.9--I10	0.47	I10--K29.5	0.46	J98--E78	0.48
	E78--K76.0	0.38	L30.9--E78	0.39	I10--K29.5	0.45	L30.9--E78	0.47	H26--I10	0.46	E78--I49	0.47
	L30.9--I10	0.37	E78--N92	0.39	E04--I10	0.44	E04--I10	0.45	L30.9--I10	0.45	E78--I50.9	0.46
	L30.9--E78	0.36	I10--N92	0.39	J06--I10	0.42	E78--I63	0.44	E78--H26	0.45	L30.9--I10	0.45
	B18.1--K73	0.35	I10--K29.5	0.39	J06--E78	0.42	N39.0--E78	0.43	L30.9--E78	0.45	L30.9--E78	0.44
	I10--K29.5	0.32	E78--K29.5	0.39	B18.1--K73	0.42	N39.0--I10	0.42	E78--M81	0.44	N39.0--I10	0.44

TableS4. The node degree of the burst disease in different age groups in male patients with type 2 diabetes, related to Figure 3.

ICD-10 Code	<40 yrs	40~49 yrs	50~59 yrs	60~69 yrs	70~79 yrs	≥80 yrs
E04	2	4	11	15	14	10
E11.3	4	5	13	12	8	2
E11.4	1	2	9	13	11	7
E11.5	0	1	8	10	7	3
E11.7	0	1	8	10	7	0
E78	10	23	50	81	71	57
H26	3	4	8	15	20	17
I10	11	21	51	84	74	59
I25	3	8	18	38	39	43
I49	0	2	5	11	16	20
I63	1	3	8	16	20	25
I70	0	1	9	18	16	14
J31	4	4	9	16	12	9
J42	0	0	1	4	7	13
K29.5	4	5	17	30	25	21
M13	0	0	4	10	16	15
M81	0	0	0	5	5	11

TableS5. The node degree of the burst disease in different age groups in female patients with type 2 diabetes, related to Figure 3.

ICD-10 Code	<40 yrs	40~49 yrs	50~59 yrs	60~69 yrs	70~79 yrs	≥80 yrs
E04	5	7	18	34	21	11
E11.4	1	1	7	16	15	6
E78	14	17	59	89	79	62
G47.9	0	2	4	11	8	8
H16.22	0	0	5	13	11	6
H26	0	1	7	24	28	21
I10	13	19	57	89	78	65
I25	0	2	16	48	48	51
I49	0	1	6	16	14	20
I50.9	0	0	2	8	11	21
I63	0	0	5	23	27	30
I70	0	0	7	14	14	10
J31	5	5	18	34	21	9
J42	0	0	0	5	6	12
J98	2	2	2	5	5	12
K05.3	0	0	3	9	9	4
K21	0	1	4	11	8	4
K29.5	4	7	31	52	39	31
K76.0	3	2	7	16	10	4
M13	0	0	11	26	25	20
M25.5	0	2	4	10	9	6
M48.9	0	0	6	13	12	7
M51.2	0	0	6	15	14	7
M54	2	2	4	10	8	5
M81	0	0	6	26	29	27
N18	3	3	6	8	10	16
N39.0	1	2	4	10	9	10

TableS6. Alterations in the network metrics after deletion of specific nodes in the multimorbidity network of male patients with type 2 diabetes, related to the STAR Methods.

Networks	Edges (PC)	Density (PC)	AD (PC)	AWD (PC)	AHC (PC)	Connected
Original network	697	0.081	10.561	2.068	0.101	TRUE
Delete E04	668 (-4.161%)	0.078 (-2.686%)	10.198 (-3.429%)	2.001 (-3.254%)	0.101 (-0.728%)	TRUE
Delete E11.4	677 (-2.869%)	0.080 (-1.375%)	10.336 (-2.128%)	2.023 (-2.195%)	0.101 (-0.379%)	TRUE
Delete E78	591 (-15.208%)	0.069 (-13.904%)	9.023 (-14.561%)	1.720 (-16.849%)	0.099 (-1.885%)	TRUE
Delete H26	670 (-3.874%)	0.079 (-2.395%)	10.229 (-3.140%)	2.003 (-3.171%)	0.101 (-0.470%)	TRUE
Delete I10	582 (-16.499%)	0.068 (-15.215%)	8.885 (-15.862%)	1.697 (-17.970%)	0.094 (-6.744%)	FALSE
Delete I25	632 (-9.326%)	0.074 (-7.931%)	9.649 (-8.634%)	1.871 (-9.553%)	0.100 (-1.152%)	TRUE
Delete I49	665 (-4.591%)	0.078 (-3.123%)	10.153 (-3.863%)	1.989 (-3.830%)	0.101 (-0.626%)	TRUE
Delete I63	655 (-6.026%)	0.077 (-4.580%)	10.000 (-5.308%)	1.959 (-5.298%)	0.098 (-2.871%)	FALSE
Delete I70	660 (-5.308%)	0.078 (-3.852%)	10.076 (-4.586%)	1.978 (-4.347%)	0.101 (-0.530%)	TRUE
Delete J31	663 (-4.878%)	0.078 (-3.415%)	10.122 (-4.152%)	1.992 (-3.707%)	0.100 (-1.574%)	FALSE
Delete J42	678 (-2.726%)	0.080 (-1.229%)	10.351 (-1.983%)	2.034 (-1.654%)	0.101 (-0.120%)	TRUE
Delete K29.5	640 (-8.178%)	0.075 (-6.765%)	9.771 (-7.477%)	1.920 (-7.170%)	0.099 (-2.152%)	FALSE
Delete K76.0	670 (-3.874%)	0.079 (-2.395%)	10.229 (-3.140%)	2.010 (-2.796%)	0.100 (-1.473%)	FALSE
Delete M13	670 (-3.874%)	0.079 (-2.395%)	10.229 (-3.140%)	2.011 (-2.771%)	0.101 (-0.319%)	TRUE
Delete M81	677 (-2.869%)	0.080 (-1.375%)	10.336 (-2.128%)	2.034 (-1.655%)	0.101 (-0.164%)	TRUE
Delete N18	674 (-3.300%)	0.079 (-1.812%)	10.290 (-2.562%)	2.001 (-3.236%)	0.101 (-0.562%)	TRUE

PC: Percentage Change

AD: Average Degree

AWD: Average Weighted Degree

AHC: Average Harmonic Centrality

TableS7. Alterations in the network metrics after deletion of specific nodes in the multimorbidity network of female patients with type 2 diabetes, related to the STAR Methods.

Networks	Edges (PC)	Density (PC)	AD (PC)	AWD (PC)	AHC (PC)	Connected
Original network	868	0.084	12.056	2.431	0.106	TRUE
Delete E04	815 (-6.106%)	0.080 (-4.784%)	11.399 (-5.449%)	2.301 (-5.328%)	0.106 (-0.865%)	TRUE
Delete E11.4	844 (-2.765%)	0.083 (-1.395%)	11.804 (-2.085%)	2.381 (-2.030%)	0.106 (-0.316%)	TRUE
Delete E78	748 (-13.825%)	0.074 (-12.611%)	10.462 (-13.222%)	2.046 (-15.821%)	0.102 (-4.593%)	FALSE
Delete H26	828 (-4.608%)	0.082 (-3.265%)	11.580 (-3.941%)	2.332 (-4.054%)	0.106 (-0.546%)	TRUE
Delete I10	750 (-13.594%)	0.074 (-12.377%)	10.490 (-12.99%)	2.050 (-15.659%)	0.104 (-2.722%)	TRUE
Delete I25	795 (-8.410%)	0.078 (-7.120%)	11.119 (-7.770%)	2.220 (-8.663%)	0.105 (-1.154%)	TRUE
Delete I49	827 (-4.724%)	0.081 (-3.382%)	11.566 (-4.057%)	2.334 (-3.982%)	0.105 (-1.450%)	FALSE
Delete I63	819 (-5.645%)	0.081 (-4.316%)	11.455 (-4.985%)	2.307 (-5.078%)	0.104 (-2.602%)	FALSE
Delete I70	839 (-3.341%)	0.083 (-1.980%)	11.734 (-2.665%)	2.369 (-2.532%)	0.106 (-0.379%)	TRUE
Delete J31	820 (-5.530%)	0.081 (-4.199%)	11.469 (-4.869%)	2.315 (-4.765%)	0.105 (-1.582%)	FALSE
Delete J42	854 (-1.613%)	0.084 (-0.227%)	11.944 (-0.925%)	2.412 (-0.784%)	0.106 (-0.043%)	TRUE
Delete K29.5	796 (-8.295%)	0.078 (-7.003%)	11.133 (-7.654%)	2.232 (-8.186%)	0.104 (-2.296%)	FALSE
Delete K76.0	840 (-3.226%)	0.083 (-1.863%)	11.748 (-2.549%)	2.374 (-2.319%)	0.106 (-0.518%)	TRUE
Delete M13	824 (-5.069%)	0.081 (-3.732%)	11.524 (-4.405%)	2.323 (-4.417%)	0.105 (-1.434%)	FALSE
Delete M81	821 (-5.415%)	0.081 (-4.083%)	11.483 (-4.753%)	2.316 (-4.721%)	0.106 (-0.535%)	TRUE
Delete N18	848 (-2.304%)	0.084 (-0.928%)	11.860 (-1.621%)	2.384 (-1.926%)	0.106 (-0.348%)	TRUE

PC: Percentage Change

AD: Average Degree

AWD: Average Weighted Degree

AHC: Average Harmonic Centrality

TableS8. Alterations in the hub diseases after random deletion of multimorbidity network's edges, related to the STAR Methods.

Networks	Edges	Deleted edges	Times	Nodes with the top 10 PageRank values
Original male network	697	0%		I10, E78, K29.5, I25, I63, J31, I70, E04, K76.0, I50.9
			1	I10, E78, K29.5, I25, I63, J31, I70, K76.0, I50.9, E04
Male network-1	690	1%	2	I10, E78, K29.5, I25, I63, J31, E04, I70, I50.9, I49
			3	I10, E78, K29.5, I25, I63, J31, I70, E04, I50.9, I49
			1	I10, E78, K29.5, I25, I63, J31, E04, I50.9, I70, I49
Male network-2	676	3%	2	I10, E78, K29.5, I25, I63, J31, I70, E04, I50.9, I49
			3	I10, E78, K29.5, I25, I63, J31, K76.0, I70, E04, I50.9
			1	I10, E78, K29.5, I25, I63, J31, I70, K76.0, E04, I49
Male network-3	662	5%	2	I10, E78, K29.5, I25, I63, J31, K76.0, E04, I70, I50.9
			3	I10, E78, K29.5, I25, I63, J31, E04, I70, I50.9, K76.0
Original female network	868	0%		E78, I10, K29.5, I25, E04, I63, J31, M13, I49, I50.9
			1	E78, I10, K29.5, I25, E04, I63, J31, M13, I49, I50.9
Female network-1	859	1%	2	E78, I10, K29.5, I25, I63, E04, J31, M13, I49, I50.9
			3	E78, I10, K29.5, I25, J31, I63, E04, M13, I49, M81
			1	E78, I10, K29.5, I25, I63, E04, J31, M13, M81, I49
Female network-2	842	3%	2	I10, E78, K29.5, I25, E04, I63, J31, M13, I49, I50.9
			3	E78, I10, K29.5, I25, I63, J31, E04, M13, I49, M81
			1	E78, I10, K29.5, I25, J31, I63, E04, M13, I49, I50.9
Female network-3	825	5%	2	I10, E78, K29.5, I25, J31, I63, E04, M13, M81, I50.9
			3	I10, E78, K29.5, I25, E04, J31, I63, M13, I49, M81
